# Elucidating the Beta-Diversity of the Microbiome: from Global Alignment to Local Alignment

**DOI:** 10.1128/mSystems.00363-21

**Published:** 2021-08-17

**Authors:** Xiaoquan Su

**Affiliations:** a College of Computer Science and Technology, Qingdao University, Qingdao, China; b Single-Cell Center, Qingdao Institute of BioEnergy and Bioprocess Technology, Chinese Academy of Sciences, Qingdao, China

**Keywords:** microbiome, beta-diversity, distance metrics, search engine, local alignment

## Abstract

Quantitative comparison among microbiomes can link microbial beta-diversity to environmental features, thus enabling prediction of ecosystem properties or dissection of host-microbiome interaction. However, to compute beta-diversity, current methods mainly employ the entire community profiles of taxa or functions, which can miss the subtle differences caused by low-abundance community members that may play crucial roles in the properties of interest. In this work, I review the distance metrics and search engines that we developed to match microbiomes at a large scale based on whole-community-level similarities, as well as their limitations in tackling the microbiome changes caused by less abundant community features. Then I propose the concept of microbiome “local alignment,” including an algorithm to measure microbiome similarity on specific fractions of biodiversity and an indexing strategy for rapidly fetching microbiome local-alignment matches from the data repository.

## COMMENTARY

Beta-diversity analysis quantifies the similarity or distance between microbiome pairs; on the basis of beta-diversity analysis, we can link the overall taxonomic or functional diversity pattern to environmental features (1) and then predict the ecosystem properties or host healthy states (2–4). Here, I summarize the algorithms and tools that we have developed for analyzing and unitizing the whole-community-level (i.e., “global”) similarities on large-scale microbiome data sets and deliver our perspective on the “local alignment” strategy that matches microbiomes by a specific subset of taxa that contribute to the properties of interest.

## SIMILARITY MEASUREMENT FOR MICROBIOMES

An accurate and reliable similarity or distance metric among microbiomes is the basis for deducing the microbial beta-diversity. Statistical or geometry approaches like Bray-Curtis, Jaccard, and Jensen-Shannon divergence calculate such distances mainly by counting the overlapped components. However, omission of the inherent relationships among community members (e.g., operational taxonomic units for 16S rRNA amplicons or species for shotgun metagenomes) can lead to unexpected, erroneous beta-diversity patterns. To tackle this issue, we introduced the Meta-Storms scoring algorithm that parses the similarity of two microbiomes by considering the evolutionary hierarchy of microbes based on a weighted reference phylogeny tree (5). It not only improves the comprehensiveness of comparison by integrating additional biological contexts but also reduces the inaccuracy caused by the sparse distribution of microbes (e.g., microbiomes collected from distinct ecosystems may lack adequate common components for comparison) (6).

On the other hand, a phylogeny-based algorithm such as Meta-Storms requires all community members are mapped to definite leaf nodes in a reference tree; however, profiles inferred from metagenomic shotgun sequences always carry unidentified or unclassified annotations. To solve this problem, we then proposed the Dynamic Meta-Storms algorithm (7), by locating the unclassified species to the virtual nodes in the phylogeny tree via their higher-level taxonomy. Usually, the tree-like algorithm is well defined by a recursive posttraversal process of a binary tree. However, since the microbial phylogeny tree has been greatly expanded by newly sequenced and annotated species, the overall computing time for the pairwise distance matrix becomes unacceptable, especially for studies with thousands of samples. Hence, optimizations including nonrecursive transformation and memory recycling were performed in Meta-Storms and Dynamic Meta-Storms to improve the efficiency of computing and memory resource (8). Coupled with parallel computing on a multicore CPU (central processing unit) or a GPU (graphics processing unit), our implementations accomplished the pairwise comparison of 100,000 metagenomes within a few hours on a single desktop computer, enabling beta-diversity analysis on a much broader scale.

## MICROBIOME SEARCH ENGINE ENABLES THE GLOBAL MATCH IN MICROBIOME DATA SPACE

Over the past years, the number of sequenced microbiomes has grown exponentially. While big data introduces a plethora of opportunities to uncover biological principles hidden under biodiversity surveys, new challenges have emerged, such as the extremely high data volume (9). One key demand and bottleneck has been relating newly sampled microbiomes to existing data. Thus, we developed a Microbiome Search Engine (MSE) for rapid search of query microbiomes against a database of microbiomes, on the whole-community level (10). Basically, with a given query community, MSE compares it against a data repository and returns top hits with highest Meta-Storms similarity in real time (e.g., <0.5 s per query in 1 million samples). This allows interpreting the property of the query based on meta-data of the matches. Moreover, by placing each individual sample under the context of the numerous microbiomes produced so far, MSE provides a bird’s-eye view on the historical development of global microbiome surveying efforts. For example, tracking the 8-year dynamics of search-based microbiome novelty score (MNS) (which evaluates the overall compositional uniqueness of a microbiome compared to its top hits in a database) for more than 100,000 samples from various habitats, we were able to define the “search boundary effect” of human microbiomes (11). Specifically, the structural novelty of human microbiomes, but not environmental ones, is approaching saturation and likely bounded. More importantly, exploring the ability to quantitatively assess microbiome “novelty” or “uniqueness” via MNS, we introduced a search-based strategy for multiple disease detection and classification (12). In this method, MSE detects unhealthy samples via their outlier novelty versus a database of samples from healthy subjects and then identifies the specific disease type by comparing these to samples from patients. We showed that accuracy and efficiency of such MSE-based disease diagnosis outperform traditional machine learning approaches. These findings highlight the promise of microbiome big-data-based diagnosis as well as “data-driven” research strategies in microbiome science.

## LOCAL ALIGNMENT FOR MICROBIOME FRACTIONS

**FIG 1:**
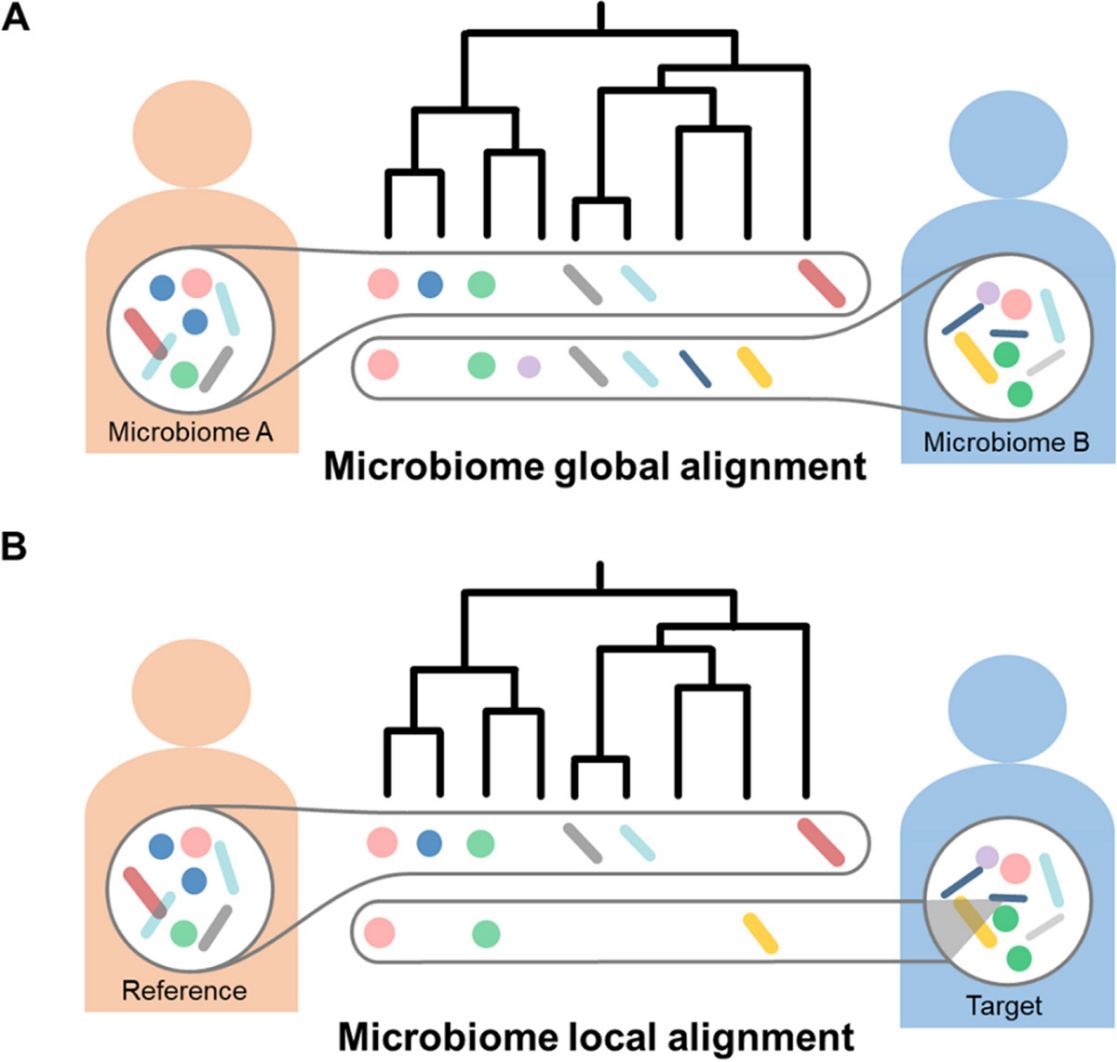
Two scenarios of microbiome comparison. (A) The end-to-end comparison of sample pairs employs whole-community-level information (i.e., “global alignment”). (B) The “local alignment” of microbiomes matches only a partial fraction of taxa that are of interest.

Usually, beta-diversity is measured by end-to-end comparison of microbiome pairs (Fig. 1A) using distance metrics like Meta-Storms, UniFrac (13), Bray-Curtis, etc. The beta-diversity-based status identification and classification relies on an assumption that most members of the community, or at least the highly abundant members, are associated with the status of interest, e.g., samples in disease group exhibit a significant compositional distinction to healthy controls (e.g., permutational multivariate analysis of variance [PERMANOVA] or analysis of similarity [ANOSIM] test *P* value of <0.01 on pairwise distances). Although previous studies have shown such beta-diversity patterns exist in many diseases such as inflammatory bowel disease (14) and colorectal cancer (15), in other cases like type 1 diabetes (16) and autism spectrum disorder (17), only a small part of signature taxa play crucial roles that can be determined by statistical tests (18) or supervised machine learning (19) but are missed by the end-to-end comparison at the whole-community level. Thus, there is an intensive need to match only the “biomarker” fractions of interest (denoted as “target”) against whole microbiomes (denoted as “reference”; Fig. 1B), just like a “local alignment” of amplified DNA fragments to the reference full-length 16S rRNA genes. Intuitively, such subcommunity-level similarity can be derived by extracting the identical features as the target from the reference and then compared it to the target. However, several issues should be appropriately covered in algorithm design and implementation. Since microbiome profiles are highly diverse and sparse across habitats (20) or cohorts (21), it is possible that a reference microbiome shares few exactly identical fractions with a target. Here, the similarity cannot be simply set as zero, and taxa with very close taxonomy or metabolic functions to the target or belong to the same guild (22) that work consistently and coherently with the target, can be considered “approximate members.” Notably, contributions of such “approximate members” should be weighted by their phylogenetic or functional distances to the “exact members.” On the other hand, however, once the “approximate members” are added for comparison, relative abundance of “exact members” will be diluted, leading to a reduction of similarity between reference and target. Therefore, for microbiome local alignment, selecting and extracting the fraction of community members from the reference microbiomes for the comparison to the target is of utmost importance.

## INDEXING STRATEGY FOR FAST FETCH OF LOCAL-ALIGNMENT HITS

Once the microbiome “local alignment” algorithm is clearly defined, suspected unhealthy microbiomes can be detected from a repository by matching with specific disease biomarkers. An exhaustive screening that compares the target fractions to all samples is a straightforward way, but it is time-consuming when the database is huge. Currently, there are two types of indexing strategies available for accelerating the microbiome search, (i) a static partitions index that groups database into subcategories sorted by structural features, e.g., Microbiome Search Engine v1.0 (5) or Meta-Prism (23); (ii) a dynamic index based on the dimension reduction of microbial profiles employed by Microbiome Search Engine 2 (10). Both of the approaches depend on the preprocessing of the entire collection of reference samples in the database construction step in order to rapidly fetch the candidate hits in the subsequent query step. Nevertheless, as the “local alignment” only takes partial community from the reference, and the range of community members relies on the specific query target (e.g., biomarkers for diseases), unified and universal indices designed for end-to-end match are not suitable for the “local alignment” scenario. A potential indexing solution to promote the speed of microbiome local-alignment can learn from the FM-index of Bowtie 2 (24) or the USEARCH algorithm (25) that were originally designed for nucleotide sequence mapping in which the target community fraction serves as a short query DNA read and the microbiomes are treated as the reference long genome sequences.

## CONCLUSION

Beta-diversity is a fundamental property of microbiomes. Highly efficient microbiome comparison, not just at the “global” level but at the “local” level, can elucidate microbial beta-diversity with higher precision and flexibility, thus contributing to in-depth comprehension and efficient utilization of microbiomes.
